# Parenting Style of Elderly Parents and Its Impact on Adolescent Behavior: A Case Report

**DOI:** 10.7759/cureus.68503

**Published:** 2024-09-03

**Authors:** Salma S, Aruna Kaki, Senthil Rajaram Manoharan, Madhusudan T, Arul Saravanan R

**Affiliations:** 1 Department of Psychiatry, SRM Medical College Hospital and Research Centre, Chennai, IND; 2 Department of Psychiatry, Huntsville Hospital, University of Alabama at Birmingham School of Medicine, Huntsville, USA

**Keywords:** elderly parents, family-focused therapy, parenting styles, covid-19 pandemic, conduct disorder

## Abstract

Adolescence is a critical transition period between childhood and adulthood. They experience biological, emotional, and social changes and require constant affection, support, and supervision during this period. Adolescents often face stressors from various sources, which can exacerbate behavioral issues. A 13-year-old boy, born 15 years after marriage by in vitro fertilization (IVF) to parents in their late 40s, was brought to the outpatient department. He had presented with complaints of getting angry, stealing money, lying, and threatening his parents for his demands for the past six months. His behavior intensified during the COVID-19 pandemic due to social isolation and prolonged home confinement. His parents said that lately, he has also gotten difficult to manage at home. He was diagnosed with conduct disorder. Despite initial management with behavioral therapy, the boy experienced frequent exacerbations of symptoms. Further assessment identified parental behavior as a contributing factor to the child's conduct disorder. Interventions incorporating family-focused therapy (FFT) and modifications in parenting techniques were implemented, resulting in an extended period of behavioral remission. Parenting style plays an instrumental role in defining the positive and negative outcomes a child will experience. Hence, the parents were counseled and psycho-educated about effective parenting. This case underscores the crucial role of parenting styles in influencing adolescent behavior and highlights the importance of family-centered interventions in managing behavioral problems during adolescence.

## Introduction

Adolescence is a vital period characterized by rapid physical, mental, and social development. Poor mental health problems can lead to risk-taking behaviors, substance use, and greater exposure to violence. Numerous factors have an impact on the mental well-being of adolescents, such as family dynamics, educational environment, and peer interactions [1]. Parents, being the closest individuals in adolescent's lives, can significantly impact their personality, mental health, and self-esteem through their parenting styles. Parenting approaches also play a crucial role in shaping an adolescent's coping mechanisms, temperament, and social skills. The idea of positive parenting emphasizes building parent-child relationships on affection, support, and communication and also setting limits, norms, and defining consequences [2]. This case report focuses on the intersection of elderly parenting and adolescent behavioral issues, particularly as these dynamics are influenced by the unprecedented challenges of the coronavirus disease 2019 (COVID-19) pandemic. The impact of COVID-19 on children, adolescents, and their families has been unprecedented. For adolescents, the pandemic has led to school closures, reduced social interactions with peers and family, and limited outdoor activities, while parents have experienced mental health issues, job losses, bereavement, and increased responsibilities. Thus, parenting becomes more crucial [3]. Through this case, we explore not only the direct effects of parenting styles on adolescent behavior but also the broader implications for developing effective, culturally sensitive interventions that can be applied in similar contexts.

## Case presentation

A 13-year-old boy in seventh grade was brought to the outpatient department by his parents with complaints of behavioral issues for the past year. In the last six months, his behavior has worsened, leading to violations of societal norms. His parents reported that he frequently became angry, lied, and stole money from home. He even threatened them when his demands were not met. The onset of these behaviors was insidious, with a continuous and deteriorating course.

Initially, his requests were relatively modest, such as asking for snacks, buying fancy clothes, some pocket money, and permission to go out with his friends. However, because of the family's financial constraints, his parents found it challenging to fulfill these requests. They often had to deny him and insisted that he focus on his studies and maintain discipline. The parents began to impose strict rules regarding his academics, behavior, and daily routines, wanting him to adhere to them firmly. Following this, there was a progressive change in his behavior.

He began forming new friendships with older children and adults in the neighborhood, most of whom were school dropouts and unemployed. He frequently engages in fights or bullies other children and disobeys his parents' words. Eventually, he started stealing money from home, roaming around with them, and even getting a tattoo on his forearm and piercing his ear to fit into this peer group. His mood was euthymic but irritable at times. His biological functions were regular, although his interest in studies decreased, and he started avoiding homework.

His parents frequently discussed their personal beliefs and the significance of upholding family values with him. However, as time passed, he grew averse to their advice and became aggressive when questioned. His behavioral issues intensified during the COVID-19 pandemic due to limited social interaction, causing even greater challenges for his parents in managing him at home. Recently, there was a further decline in his behavior as he began threatening his parents with sharp objects to fulfill his demands. Recognizing the severity of the situation, the parents sought professional help.

There is no history suggestive of organicity, intellectual disability disorder, autism spectrum disorder, or attention-deficit/hyperactivity disorder (ADHD). There is no history of decreased need for sleep, excessive speech, reduced energy levels, or persistent crying spells. There is also no history of substance use. This is his first psychiatric consultation, with no medical comorbidities. There is no family history of psychiatric illness.

Exploration of personal history and family atmosphere revealed that the mother's pregnancy was achieved through in vitro fertilization (IVF) in her 40s, after 15 years of marriage. The antenatal, perinatal, and postnatal periods were uneventful. He was exclusively breastfed for up to four months, followed by mixed feeding due to low production of breast milk, and weaning started at seven months. His developmental milestones were appropriate for his age. His scholastic history includes studying in a normal school, starting at the age of four, with average academic performance that has currently declined over the past year. His attendance has been regular, and he has made adequate peer group adjustments with good relations with authorities and no disciplinary actions against him. His leisure activities included playing outdoor games and watching cartoons. Currently, he watches YouTube videos, plays video games, and mostly goes out with his friends. His general temperament and personality attributes showed that the child was active and energetic but had stubborn and aggressive tendencies and quickly adapted to new experiences. The family mainly survives on pension money to make ends meet. Due to the significant generational gap, his parents have less time to interact with him. Patterns of parental functioning include rigidity, strict discipline, protectiveness, intolerance to deviance, and high expectations for the child.

On examination, he was alert and cooperative, with a tattoo on his forearm and one ear pierced; no abnormal movements were observed. A physical examination, including a neurological assessment, revealed no abnormalities. An intelligent quotient (IQ) assessment was conducted and found to be within normal limits. Based on the International Classification of Diseases, Tenth Revision (ICD-10) criteria, the patient was diagnosed with conduct disorder, characterized by persistent behavior that violates societal norms and the rights of others. The key behaviors observed in this case, such as aggression, deceitfulness, and serious rule violations, fit the diagnostic profile. Differential diagnoses considered included ADHD and oppositional defiant disorder (ODD). However, the absence of core symptoms such as hyperactivity and persistent defiance, coupled with the presence of more severe behaviors like theft and aggression, supported a diagnosis of conduct disorder.

As the primary concern was the child's behavioral issues, non-pharmacological management was considered. The child received behavioral therapy aimed at addressing his maladaptive behavior and improving his coping strategies. Therapy sessions were conducted once a week, each lasting 30 minutes. However, frequent flare-ups continued. Upon a detailed reassessment, it was found that the nature of parental engagement significantly contributed to the persistence of his behavioral issues. As a result, adjustments were made to the therapeutic approach. Parents were psycho-educated on how to modify their parenting style to support their son, incorporating elements of family-focused therapy (FFT). This therapy targeted maladaptive family interactions and included emotional regulation training, setting clear rules, and enhancing parental involvement. Six sessions of FFT were conducted, one per week, each lasting 45 minutes (Table 1). As a result, the child's behavioral issues began to settle down, and family interactions improved. Adapting these modified parenting techniques led to longer periods of remission and reduced the frequency and intensity of his behavioral outbursts.

**Table 1:** Details of the family-focused therapy session conducted

Session	Core Components	Objectives	Behavioral Modifications Observed in Follow-up
Session 1	Psychoeducation	Identification of relevant stressors. Discussion about conduct disorder. Analyzing the effect of the disorder on regular family functioning and coping skills followed.	Better understanding of the behavioral problems, creating positive expectations and increased motivation.
Session 2	Psychoeducation	Discussion on ineffective parenting practices, association with deviant peers, and declined academic performance—special emphasis on regulating family routines and enhancing communication among them.	Parents understood that harsh punishments and oppressive demands end up exacerbating the maladaptive behavior of the child. Child understood about his problem behavior and its impact on family.
Session 3	Communication enhancement training	To enhance family connections by breaking unhealthy patterns of behavior, redistributing the priority between praise and criticism, raising empathy and active listening skills.	Quality of the parent-child relationship improved. A change in the parenting style was noticed.
Session 4	Communication enhancement training	Expressing unfavorable feelings about particular actions, asking politely for behavioral adjustments from one another.	Better communication within them started to express their feelings.
Session 5	Problem-solving	To identify specific problems, to teach the problem-solving approach using examples.	Child identified his problems and showed interest in problem analysis and resolution. This resulted in improvement in the behavioral issues.
Session 6	Problem-solving	Creating solutions as a group, evaluating the benefits and drawbacks of each, and building an implementation strategy.	Reduced family stress and improved functioning.

## Discussion

This case report highlights the complex relationship between parenting styles and adolescent behavior, especially in the context of elderly parents raising their children. Here, the parents had specific expectations from their child, shaped by their own experiences and cultural background. The child's modern-day environment, marked by advanced technology, social media, and diverse peer interactions, clashed with his parent's expectations. The boy perceived his parent's firm rules and high expectations as restrictive, and he sought autonomy and acceptance outside the home, which led to delinquent behavior.

Parenting style refers to the combination of the parents' attitudes, behaviors, and the emotional environment they create during the upbringing of their children. Baumrind (1966) identified three distinct parenting styles based on the dimensions of responsiveness and demandingness: permissive, authoritative, and authoritarian. The authoritarian parenting style is characterized by controlling relationships, one-way communication, and restricted autonomy, while authoritative parenting provides the appropriate level of independence and emotional support. Permissive parents, although warm and nurturing, set minimal expectations and provide more autonomy. Later, Maccoby and Martin (1983) introduced a fourth style, neglectful or uninvolved. Uninvolved parents provide basic needs but remain detached [4].

In India, parenting styles are heavily shaped by collectivistic cultural values, prioritizing the interests of the family or community over individual interests. It is widely believed that children are representations of their parents; thus, a child's behavior is expected to align with family values. As a result, Indian cultures often favor an authoritarian parenting style compared to those in Western cultures [5]. In this case, the parents also adhered to a conservative, authoritarian style, which reflects their upbringing and cultural values. Excessive controlling behavior with strict rules is one of the factors attributing to stress, aggression, and behavioral problems in the child. Similarly, in one study, a detailed analysis of parenting styles revealed that the authoritarian maternal style of parenting is linked to delinquent outcomes compared to the authoritative style [6]. Another study also showed a significant positive correlation between increased aggression and authoritarian parenting, further emphasizing the potential negative behavioral outcomes associated with this parenting style [7]. In various studies conducted in India, the authoritative parenting style consistently showed the most positive outcomes in children with better emotional intelligence, high self-esteem, and coping strategies [5,8,9].

In this case, the child was diagnosed with conduct disorder. It can be due to various factors, including his temperament, parental expectations, financial constraints, and limited social interactions exacerbated by the COVID-19 pandemic.

The COVID-19 pandemic drastically changed the daily lives of families, causing significant stress. Many parents had to work from home while managing their children or balancing work at essential jobs with finding childcare when schools closed. Additionally, concerns about family health, finances, and an uncertain future added pressure. As parents are stressed over a long period, they experience parental burnout, which has serious effects on both them and their children. These new challenges increased the risk of negative outcomes for both parents and children. Social distancing and isolation during the pandemic made parenting more challenging. Overall, many parents reported that their emotions, particularly negative ones, affected their ability to parent during this challenging time [10,11].

Children with conduct disorders have a greater chance of developing mental health issues in adulthood, including antisocial personality disorder. Behavioral therapy was given, focusing on setting and monitoring behavioral goals, rewarding the positive, and punishing the negative ones. It aims to improve social cognition and reduce impulsivity [12]. In a recent study, boys with conduct disorder or ODD showed moderate improvement in behavior and quality of life when receiving social competence training compared to group play, with parents and clinicians noting significant benefits [13].

Effective parent-child communication reduces risky behaviors and promotes healthier adjustments. Supportive relationships between parents and adolescents act as a protective buffer against stress and negative influences [14]. FFT addresses negative interactions caused by insecure family attachment bonds. It aims to build secure attachments. Parents are taught to be supportive and responsive, while children are encouraged to trust and express their needs. This approach reduces conflicts, alleviates stress, and improves resilience [15]. In our case, FFT concentrated on modifying ineffective parenting practices and adapting the parenting style from authoritarian to authoritative. This allowed the parent and child to form a close bond. Each parenting style has its own impact on a child's behavior and well-being. The permissive style will lead to impulsiveness and selfishness. A neglectful parenting style would result in a child with inadequate coping skills and dysfunctional emotional regulation. Since other parenting styles would not have led to the desired outcome, we recommended the authoritative style in our case. As a result, the child's problematic behavior decreased, and he became more responsible through improved emotional regulation.

## Conclusions

This case highlights the psychological impact of parenting approaches, particularly in the context of elderly parents raising an adolescent. It highlights the importance of a balanced parenting style in supporting healthy adolescent development, especially during developmental stages. Addressing the root causes embedded in family dynamics and promoting positive parenting strategies, apart from usual treatment, helps improve developmental and behavioral outcomes in adolescents.

## References

[1] Peng B, Hu N, Yu H, Xiao H, Luo J (2021). Parenting style and adolescent mental health: the chain mediating effects of self-esteem and psychological inflexibility. Front Psychol.

[2] Lorence B, Hidalgo V, Pérez-Padilla J, Menéndez S (2019). The role of parenting styles on behavior problem profiles of adolescents. Int J Environ Res Public Health.

[3] Karki U, Dhonju G, Raj Kunwar A (2020). Parenting during the COVID-19 pandemic. JNMA J Nepal Med Assoc.

[4] Jinan N, Yusof NAM binti M, Vellasamy V (2022). Review of parenting styles and their impact on the adolescent's self-esteem. Int J Acad Res Prog Educ Dev.

[5] Sahithya BR, Manohari SM, Vijaya R (2019). Parenting styles and its impact on children—a cross cultural review with a focus on India. Ment Health Relig Cult.

[6] Moitra T, Mukherjee I (2010). Does parenting behaviour impacts delinquency? A comparative study of delinquents and non-delinquents. Int J Crim Justice Sci.

[7] Ranjana MN (2017). Parenting styles and self-esteem as predictors of aggression. IJHW.

[8] Amandeep D (2017). Emotional intelligence in relation to perceived parenting style of early adolescents. Int J Indian Psychol.

[9] Bhattacharyya P, Pradhan RK (2015). Perceived paternal parenting style and proactive coping strategies of Indian adolescents. Int J Psychol Stud.

[10] Kerr ML, Rasmussen HF, Fanning KA, Braaten SM (2021). Parenting during COVID-19: a study of parents' experiences across gender and income levels. Fam Relat.

[11] Ping Y, Wang W, Li Y (2021). Father's parenting stress, parenting styles and children’s problem behavior: the mediating role of parental burnout. Curr Psychol.

[12] National Institute for Health and Clinical Excellence (NICE) and the Social Care Institute for Excellence (SCIE) (2013). Antisocial behaviour and conduct disorders in children and young people: recognition and management. British Psychological Society.

[13] Sagar R, Patra BN, Patil V (2019). Clinical practice guidelines for the management of conduct disorder. Indian J Psychiatry.

[14] Hoskins DH (2014). Consequences of parenting on adolescent outcomes. Societies.

[15] Conradi HJ, Meuwese D, Rodenburg L, Dingemanse P, Mooren T (2023). Effectiveness and feasibility of structured emotionally focused family therapy for parents and adolescents: protocol of a within-subjects pilot study. PLoS One.

